# Methylome and Complete Genome Sequence of Parageobacillus toebii DSM 14590^T^, a Thermophilic Bacterium

**DOI:** 10.1128/MRA.00589-20

**Published:** 2020-06-18

**Authors:** E. Anne Hatmaker, Kaela B. O’Dell, Lauren A. Riley, Irenee C. Payne, Adam M. Guss

**Affiliations:** a Biosciences Division, Oak Ridge National Laboratory, Oak Ridge, Tennessee, USA; b Bredesen Center for Interdisciplinary Research and Graduate Education, University of Tennessee—Knoxville, Knoxville, Tennessee, USA; Georgia Institute of Technology

## Abstract

Here, we present the first complete genome assembly of the thermophilic bacterium Parageobacillus toebii DSM 14590^T^. The *P. toebii* DSM 14590^T^ genome consists of a 3,270,071-bp circular chromosome and a 52,989-bp native plasmid.

## ANNOUNCEMENT

*Parageobacillus* is a recently defined genus of Gram-positive, facultative thermophiles; many *Parageobacillus* species were formerly classified as *Geobacillus* species (1). Members of both *Geobacillus* and *Parageobacillus* have potential for biotechnological applications and can produce thermostable enzymes. The aerobic bacterium Parageobacillus toebii DSM 14590^T^ was isolated from hay compost in South Korea (2). The genome sequence and methylome of *P. toebii* DSM 14590^T^ will inform approaches for genetic engineering of the strain and provide resources for studying *Parageobacillus* species in general.

*P. toebii* DSM 14590^T^ was acquired from the German Collection of Microorganisms and Cell Cultures (DSMZ) and was grown in LB liquid medium with 3 g/liter beef extract at 55°C. We used the 100/G Genomic tip extraction kit and bacterial protocol (Qiagen, Valencia, CA, USA) to isolate genomic DNA from *P. toebii* DSM 14590^T^. The DNA was not sheared or size selected. Long-read sequencing of *P. toebii* DSM 14590^T^ was generated at the DOE Joint Genome Institute (JGI). A PacBio SMRTbell library was constructed and sequenced on the PacBio RS II platform (Menlo Park, CA, USA) (3). Sequencing generated 379,771 filtered subreads totaling 769,802,073 bp; PacBio filtering removes reads if the quality score is <0.75 or the length is <50 bp and trims hairpin adapters from sequences, splitting them into subreads. The reads were assembled using the Hierarchical Genome Assembly Process 3 (HGAP3) v2.3.0 (4) with default parameters.

The genome of *P. toebii* DSM 14590^T^ contains a circular chromosome 3,270,071 bp long and a circular plasmid of 52,989 bp. Long-read sequencing data provided 350× coverage when mapped back to the assembled genome and over 1,800× coverage for the plasmid, suggesting an average plasmid copy number of 5. The genome was annotated using the NCBI Prokaryotic Genome Annotation Pipeline (PGAP) (5), which predicted 3,375 chromosomal genes, including 3,253 protein-coding, 28 rRNA, and 90 tRNA genes. Additionally, the chromosome is predicted to encode four noncoding RNAs. The *P. toebii* plasmid, pDSM14590, is predicted to encode 61 genes, all of which are considered protein-coding genes.

JGI also performed DNA modification detection and motif analysis using the PacBio SMRT analysis platform v5.0.1.9585 with default parameters. Modified sites were then identified and grouped into motifs using MotifFinder (6), with motifs representing recognition sequences of active methyltransferase genes (7). One methylated motif, garaAtt, was found (100% of occurrences modified). No restriction-modification (RM) systems were predicted within the *P. toebii* DSM 14590^T^ genome, but all the necessary genes involved in the bacteriophage exclusion (BREX) system were predicted (DER53_11070 to DER53_11085, DER53_12455, and DER53_16290). Like RM systems, BREX is a methylation-based bacterial defense system wherein a nonpalindromic sequence is methylated on the genome and infection by unmethylated phage DNA is hindered, though the mechanism is not fully elucidated (8). No prophage regions were predicted within the *P. toebii* DSM 14590^T^ genome using VirSorter (9) with default parameters.

### Data availability.

The accession number for the *P. toebii* DSM 14590^T^ chromosome is CP049703, and the plasmid pDSM14590 is available under the accession number CP049704. The BioProject accession number is PRJNA455457, the BioSample accession number is SAMN09062732, and the reads have been deposited in the NCBI SRA under accession numbers SRX4823098 and SRX4823099.
